# Treatment of KRAS-Mutated Pancreatic Cancer: New Hope for the Patients?

**DOI:** 10.3390/cancers17152453

**Published:** 2025-07-24

**Authors:** Kamila Krupa, Marta Fudalej, Emilia Włoszek, Hanna Miski, Anna M. Badowska-Kozakiewicz, Dominika Mękal, Michał P. Budzik, Aleksandra Czerw, Andrzej Deptała

**Affiliations:** 1 Students’ Scientific Organization of Cancer Cell Biology, Department of Oncology Propaedeutics, Medical University of Warsaw, 01-445 Warsaw, Poland; kamila.krupa@student.wum.edu.pl (K.K.); s090695@student.wum.edu.pl (H.M.); 2 Department of Oncological Propaedeutics, Medical University of Warsaw, 01-445 Warsaw, Poland; marta.fudalej@wum.edu.pl (M.F.); anna.badowska-kozakiewicz@wum.edu.pl (A.M.B.-K.); michal.budzik@wum.edu.pl (M.P.B.); 3 Department of Oncology, National Medical Institute of the Ministry of the Interior and Administration, 02-507 Warsaw, Poland; 4 Department of Health Economics and Medical Law, Medical University of Warsaw, 02-091 Warsaw, Poland; aleksandra.czerw@wum.edu.pl; 5 Department of Economic and System Analyses, National Institute of Public Health NIH—National Research Institute, 00-791 Warsaw, Poland

**Keywords:** KRAS mutations, sotorasib, adagrasib, MRTX1133, pan-RAS inhibitors, ADT-007, RMC-9805, RMC-6236, RNAi, pancreatic cancer

## Abstract

Pancreatic cancer, specifically pancreatic ductal adenocarcinoma (PDAC), ranks among the most lethal malignancies, with a 5-year survival rate of under 10%. The most prevalent KRAS mutations occur in three hotspot residues: glycine-12 (G12), glycine-13 (G13), and glutamine-61 (Q61), leading to the constant activation of the Ras pathway, making them the primary focus in oncologic drug development. Selective KRAS G12C inhibitors (e.g., sotorasib, adagrasib) have demonstrated moderate efficacy in clinical trials; however, this mutation is infrequent in PDAC. Emerging therapies targeting KRAS G12D and G12V mutations, such as MRTX1133, PROTACs, and active-state inhibitors, show promise in preclinical studies. Pan-RAS inhibitors like ADT-007, RMC-9805, and RMC-6236 compounds provide broader coverage of mutations. Their efficacy and safety are currently being investigated in several clinical trials. A major challenge is the development of resistance mechanisms, including secondary mutations and pathway reactivation. Combination therapies targeting the RAS/MAPK axis, SHP2, mTOR, or SOS1 are under clinical investigation. Immunotherapy alone has demonstrated limited effectiveness, attributed to an immunosuppressive tumor microenvironment, although synergistic effects are noted when paired with KRAS-targeted agents. Furthermore, KRAS mutations reprogram cancer metabolism, enhancing glycolysis, macropinocytosis, and autophagy, which are being explored therapeutically. RNA interference technologies have also shown potential in silencing mutant KRAS and reducing tumorigenicity. Future strategies should emphasize the combination of targeted therapies with metabolic or immunomodulatory agents to overcome resistance and enhance survival in KRAS-mutated PDAC.

## Simple Summary

Pancreatic cancer (PC) has the highest mortality rate among all major cancers. Most pancreatic ductal adenocarcinomas (PDACs) harbor mutations in the KRAS gene, making it a primary therapeutic target. Although KRAS has long been considered “undruggable,” new research has led to the development of inhibitors that specifically target certain KRAS variants, including G12C, G12D, and G12V. Furthermore, new strategies, including RNA interference, metabolic pathway modification, proteolysis-targeting chimeras (PROTACs), and pan-RAS inhibitors, are being actively investigated in pre-clinical and clinical trials. Despite recent progress, there is still a need for more effective and personalized therapies. Moreover, resistance mechanisms and the tumor microenvironment influence the course of treatment. Combinations of drugs targeting downstream pathways or immunologic components are thought to be promising solutions for improving efficacy.

## 1. Introduction

Despite significant efforts in developing pioneering targeted therapies, pancreatic cancer (PC) currently has the highest mortality rate of all primary cancers. Unlike other gastrointestinal tumors, pancreatic adenocarcinoma is exhibiting increasing incidence. It now represents the fourth leading cause of cancer death globally and is projected to become the second leading cause by 2030. Survival rates remain extremely low; according to recent statistics, only 13% of patients survive five years after diagnosis [1]. PC is mostly diagnosed at late stages, so there is a need for a non-invasive method that could serve in differential diagnosis. A recent study showed that computed tomography and radiomics analysis could be an option [2].

The poor response to available treatments and the early deterioration in patient performance complicate the management of this disease: patients exhibit an early alteration in metabolic state with rapid weight loss, leading to increased chemotherapy toxicities [3,4]. However, recent advances in molecular-targeted therapies offer promising strategies to improve the outcomes. γ-tocotrienol can activate the Jun N-terminal kinase (JNK) apoptotic pathway, increase the synthesis of C:16 ceramide, which promotes apoptosis, and inhibit the mitogen-activated protein kinase (MAPK) and phosphatidylinositol 3-kinase/protein kinase-B (PI3K/AKT) pathways. The other isoform δ-tocotrienol altered angiogenesis by inhibiting vascular endothelial growth factor (VEGF) and matrix metalloproteinase-9 (MMP9), resulting in decreased metastatic activity. These mechanisms show that bioactive compounds from natural products may offer many benefits in treatment and prevention [5].

Prevalence is higher in industrialized countries than in developing countries, indicating that environmental factors serve as key risk factors for the disease [4]. Lack of physical activity, obesity, chronic pancreatitis, diabetes, smoking, alcohol consumption, and metabolic syndrome are also recognized as risk factors for the development of PC [6,7,8]. The connection between polycystic ovarian syndrome (PCOS) and PC is under investigation; however, a recent randomization study showed that genetically predicted PCOS is not causally associated with PC risk in Europeans [9].

Under physiological conditions, the pancreas performs both endocrine and exocrine functions. The endocrine function of the pancreas regulates metabolism in the body through the production of insulin, glucagon, and other hormones. In contrast, exocrine function primarily produces enzymes necessary for digesting fats, carbohydrates, and proteins [8,10]. Pancreatic exocrine insufficiency (PEI) manifests as nutrient malabsorption, accompanied by jaundice, unintentional weight loss, anorexia, epigastric pain, early satiety, and symptoms such as nausea, involuntary vomiting, dehydration, diarrhea, and steatorrhea; consequently, it can lead to protein–energy malnutrition, while hormonal failure most often results in the development of diabetes [8,11,12].

The American Joint Committee on Cancer (AJCC) established classifications for tumor, lymph nodes, and metastasis to stratify patients and evaluate prognosis [13]. Additionally, the National Comprehensive Cancer Network (NCCN) divided PC into a resectable, borderline resectable, locally advanced, and metastatic stages. Anatomical and biological criteria, as well as conditional factors, are critical to fully assess surgical eligibility. Preoperative evaluation should include history, physical examination, detailed chest imaging, computed tomography of the abdomen to evaluate the regional and distant lymph node metastasis and tumor contact to vessels, and lab studies with tumor markers like CA19.9. Based on the results, clinicians could accurately stage the patient [14,15].

Among patients with PC, survival rates are significantly higher for those who have undergone surgery compared to those with unresectable tumors [12]. Unfortunately, fewer than 20% of PC patients qualify for resectable surgery [16]. This low resection rate is closely associated with advanced cancer stages, tumor location, patient comorbidities, and reduced performance status [12].

Multidisciplinary treatment is the most common choice for patients diagnosed with PC and includes surgery, chemotherapy, chemoradiotherapy, and supportive care [17,18,19,20]. Unfortunately, most patients are diagnosed at an advanced or metastatic stage of the disease. The most frequent sites of distant metastases are the liver (90%), lymph nodes (25%), lungs (25%), peritoneum (20%), and bones (10–15%). In these cases, palliative chemotherapy is the only treatment option, and nearly all patients eventually relapse, requiring second-line options that offer limited choices [20,21,22].

This study aims to provide a comprehensive review of the Kirsten rat sarcoma viral oncogene (KRAS)-related treatment for patients with PC, especially pancreatic ductal adenocarcinoma (PDAC).

## 2. PDAC Molecular Subtypes

Cellular injury or damage to the pancreas can induce acinar cells to revert to an progenitor-like embryonic state, resulting in acinar-to-ductal metaplasia (ADM) and subsequently pancreatic intraepithelial neoplasia (PanIN 1–3) [23]. One of the most common histological subtypes arising from this pathway is PDAC. In contrast, tumors originating from the endocrine component are neuroendocrine tumors (NETs) [17].

PDAC can be further classified into two molecular subtypes: classical and basal-like. The classical subtype is more common, typically more differentiated, and more frequently resectable. It is often associated with the KRAS G12V mutation and higher expressions of SMAD4 and GATA6. The basal-like subtype, on the other hand, is characterized by poor differentiation, worse clinical outcomes, and a stronger association with the KRAS G12D mutation [24,25]. Despite the poorer prognosis associated with basal-like cancers, patients exhibiting this subtype had a significant inclination towards improved responses to adjuvant therapy [24].

Tumor heterogeneity remains a major challenge in developing effective treatments. Although the classical/basal-like classification is useful, mixed tumors also exist. Vimentin, a marker of epithelial-to-mesenchymal transition (EMT), is more commonly associated with the basal-like subtype, but there is possibility for presence the both vimentin-positive and vimentin-negative cells in one tumor. This can lead to increased tumor aggressiveness and worse clinical outcome [25].

## 3. KRAS Mutation—Biomolecular Introduction

A set of recurrent mutations in key oncogenes and tumor suppressor genes defines PDAC at the genetic level. Four core mutations are commonly observed: KRAS (~85%), TP53 (60–70%), CDKN2A (>50%), and SMAD4 (~50%). Additional mutations occur less frequently in genes related to epigenetic regulation (such as ARID1A, MLL2, KDM6A) and DNA damage response (such as ATM, BRCA2) [26]. At the PanIN stage, KRAS mutations typically arise early, while alterations in CDKN2A, TP53, and SMAD4 are linked to disease progression and invasive cancer. The high prevalence of KRAS mutations emphasizes the Ras signaling pathway as a central driver and therapeutic target in PDAC. Beyond genetic mutations, PDAC can also be categorized into subtypes based on histology, gene expression, and the tumor microenvironment (TME), which may affect how mutations such as KRAS influence disease behavior and treatment response [27].

### 3.1. KRAS—The Mechanism of Action in the Cancer Cell

*The KRAS* gene encodes a member of the Ras family of small GTPases, alongside its paralogs Harvey rat sarcoma viral oncogene (HRAS) and neuroblastoma rat sarcoma viral oncogene (NRAS), all of which are commonly mutated in cancer. KRAS mutations are widespread in PDAC (~85%), colorectal cancer (~45%), and lung adenocarcinoma (~30%), with lower rates in other cancers [28]. Most mutations occur at hotspot residues G12, G13, and Q61, disrupting GTPase activity and locking Ras in an active, GTP-bound state. Overall, *RAS* gene mutations are found in over 10% of human cancers. Despite their prevalence, effective targeted therapies remain lacking, and Ras mutations are often linked to poor prognosis and resistance to other treatments, making Ras a key focus in cancer research [29].

The Ras family proteins—KRAS, HRAS, and NRAS—are peripheral membrane proteins that contain a conserved G-domain, which includes the GTPase catalytic site and two switch regions that change conformation during GDP/GTP exchange. Their C-terminal regions vary, with KRAS generating two isoforms, KRAS4A and KRAS4B, through alternative splicing. Ras proteins experience post-translational modifications, such as farnesylation (in all isoforms) and palmitoylation (in HRAS, NRAS, and KRAS4A), which are essential for their membrane association and functionality. These lipid modifications anchor Ras to the plasma membrane and other cellular membranes, facilitating proper signaling activity [30].

Ras proteins function as signal transducers regulated by growth factors, cytokines, and hormone receptors. When these receptors are activated, guanine nucleotide exchange factors (GEFs) promote GDP-GTP exchange on Ras, switching it to an active state. Active, GTP-bound Ras changes shape, allowing its switch regions to bind and activate downstream effectors. Ras is inactivated when GTP is hydrolyzed to GDP, a process sped up by GTPase-activating proteins (GAPs). However, common Ras mutations at G12, G13, and Q61 disrupt GAP binding or GTPase activity, locking Ras in its active state and causing continuous, receptor-independent signaling that drives cancer progression [31].

**Figure 1:**
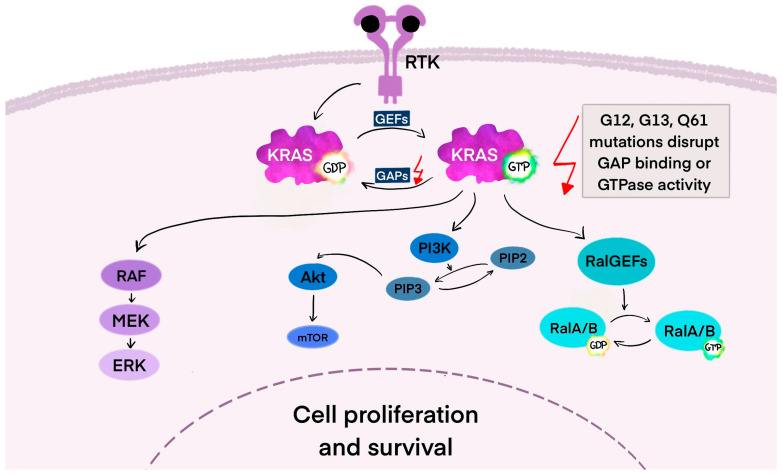
The cell regulation of KRAS protein. When these receptors are activated, guanine nucleotide exchange factors (GEFs) promote GDP-GTP exchange on Ras, switching it to an active state. It is inactivated when GTP is hydrolyzed to GDP, a process sped up by GTPase-activating proteins (GAPs). Ras mutations are commonly selected in cancer because Ras activates multiple effector pathways that promote oncogenic transformation. These include the MAPK cascade (RAF–MEK—ERK), PI3K/AKT/mTOR, and Ral guanine nucleotide exchange factors pathway [31,34,35,36,37,38]. Abbreviations: KRAS—Kirsten rat sarcoma viral oncogene; RTK—receptor tyrosine kinase; GAPs—GTPase-activating proteins; GEFs—guanine nucleotide exchange factors; GDP—guanosine diphosphate; GTP—guanosine triphosphate; RAF—rapid fibrosarcoma; ERK—extracellular signal-regulated kinase.

In its resting or “OFF” state, KRAS binds GDP and does not trigger downstream signaling. GEFs, such as Son of sevenless 1 and 2 (SOS1/SOS2), growth factor receptor-bound protein (GRB2), and RAS protein-specific guanine nucleotide-releasing factor 2 (RASGRF2), promote the exchange of GDP for GTP in response to signals like epidermal growth factor (EGF) and cytokines. Src homology-2 domain-containing protein tyrosine phosphatase (SHP2), a scaffolding protein, supports this process by stabilizing the GRB2/SOS1 complex at the cell membrane [32]. As illustrated in Figure 1, when KRAS binds GTP, it switches to the “ON” state and activates signaling pathways. To return to the “OFF” state, KRAS-GTP must be hydrolyzed to KRAS-GDP, a process that is inefficient without assistance from GAPs such as neurofibrin-1 (NF1) and p120-RasGAP protein (RASA1) [33].

Ras mutations are frequently selected in cancer because Ras activates numerous effector pathways that foster oncogenic transformation. These pathways include MAPK, PI3K/AKT/mTOR, Rho/Rac/Ral GTPases, and phospholipase C. Collectively, they regulate essential cellular processes such as proliferation, growth, survival, metabolism, motility, and gene expression. When Ras is constitutively active due to mutation, it drives continuous signaling through these pathways, providing cancer cells with a significant advantage in growth, survival, and metastasis [34].

A key pathway is the MAPK cascade (RAF–MEK—ERK), where activated KRAS initiates rapid fibrosarcoma (RAF) dimerization and phosphorylation, leading to the activation of extracellular signal-regulated kinase 1 and 2 (ERK1/2). ERK then translocates to the nucleus, activating transcription factors that drive cell cycle progression. The degree of RAF activation can vary and may influence outcomes depending on specific KRAS mutations [35]. KRAS also signals through the PI3K/protein kinase B (AKT) pathway. The activation of phosphoinositide 3-kinase (PI3K) leads to the production of phosphatidylinositol (3,4,5)-trisphosphate (PIP3) and AKT phosphorylation, promoting cell growth, survival, metabolism, and insulin response. AKT also interacts with mTOR signaling and Bcl-2 family proteins, balancing proliferation and apoptosis [36,37]. The third key RAS effector pathway involves Ral guanine nucleotide exchange factors. Like other GTPases, RalA and RalB alternate between active (GTP-bound) and inactive (GDP-bound) states. This pathway functions alongside RAS/RAF and RAS/PI3K signaling and activates the JNK pathway, promoting cell migration and proliferation [38].

### 3.2. KRAS-Mutant Cancers and Specific Codon Mutations

The reasons why KRAS mutations are more common than HRAS or NRAS mutations in adenocarcinomas are not fully understood. One possibility is that KRAS expression levels in epithelial tissues may be better suited to drive proliferation and transformation. Studies show that mutant KRAS promotes epithelial cell proliferation and tumor stemness more effectively, partly by suppressing non-canonical Wnt signaling. This suggests KRAS may have unique signaling advantages over its paralogs [39,40].

In KRAS-mutant cancers, specific codon mutations differ by tissue type. For example, G13, K117, and A146 mutations occur more frequently in colorectal cancer (CRC), while G12C is common in lung cancer, likely due to smoking-related mutagenesis. In contrast, G12D and G12V are predominant in PDAC, along with G12R, which is rare in CRC and lung cancer [41]. The reason for these tissue-specific mutation patterns remains unclear, but it may involve the context-dependent effects of each mutation on Ras signaling [42].

**Figure 2:**
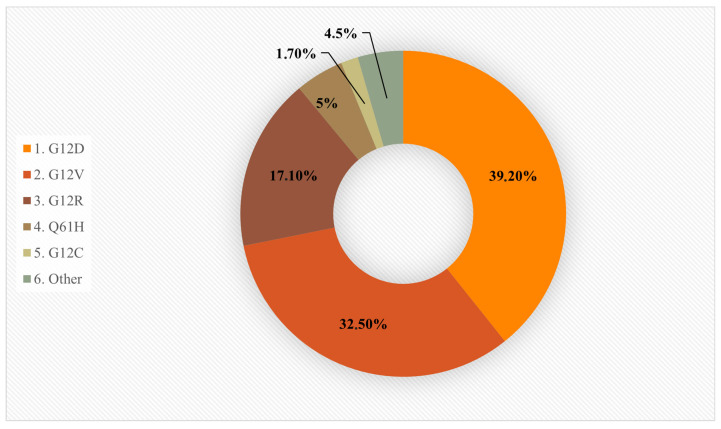
Common KRAS mutations in PDAC. Chart shows the approximate prevalence of major KRAS mutations in PDAC. The most frequent subtype is G12D (39.2%), followed by G12V (32.5%) and G12R (17.1%). Less common mutations include Q61H (5%), G12C (1.7%), and other mutations (4.5%). Based on [47].

Oncogenic KRAS mutations mainly occur at specific hotspots, with about 95% found in codons 12, 13, and 61. In PDAC, mutations are most frequently seen at codon 12, where a single amino acid change replaces glycine: G12D, G12V, G12R, and Q61H. The G12C mutant Ras occurs in only 1–3% of PDAC [43]. Less common mutations affect codons 13 and 61, with KRAS Q61H found in about 5% of cases and associated with improved survival, while KRAS G12D is linked to poorer outcomes [44,45]. These mutations enhance the affinity of KRAS for GTP and alter its shape, reducing its ability to be turned off by GAPs and making it resistant to its own intrinsic GTPase activity. Mutant KRAS may rely more on its limited intrinsic hydrolysis than on GAPs for inactivation [46]. A graphical overview of the prevalence of KRAS mutation subtypes in PDAC is presented in Figure 2 [47].

### 3.3. Common Co-Mutations

“Driver genes” refer to PC’s four most frequently mutated genes: *KRAS*, *TP53*, *CDKN2A*, and *SMAD4*. In the case of PDAC, 30–75% of patients have mutations in at least two of these four genes. Furthermore, it has been noted that the prognosis deteriorates as the number of mutations in these genes increases, particularly in patients with mutations in more than three genes [48]. Additionally, the most common genetic alterations in PDAC, which account for 70–90% of cases, are the co-occurring KRAS and TP53 mutations [49]. Another mutation that occurs alongside KRAS in PDAC is the *ERBB2* mutation, which enhances KRAS-driven traits and fosters tumor growth [50]. However, mixed outcomes have been recorded when treating ERBB2(HER2)-amplified PDAC with anti-HER2 therapy [51].

### 3.4. KRAS Wild-Type

Wild-type KRAS is found in a narrow subset of PDAC, occurring in approximately 10% of PDAC. A study of 2483 patients indicated that the most frequently mutated gene was TP53, and BRAF followed by gene amplifications in *FGF3* (3%), *ERBB2* (2.2%), *FGFR3* (1.8%), *NTRK* (1.8%), and *MET* (1.3%). A high amount of mutations altered the DNA damage repair, chromatin remodeling, and cell-cycle control pathways. Furthermore, wild-type KRAS exhibited gene fusions of *BRAF* (6.6%), *FGFR2* (5.2%), *ALK* (2.6%), *RET* (1.3%), and *NRG1* (1.3%) [52]. In exploratory analysis with genome and transcriptome characterization, the highest amount of copy number amplifications was connected with chromosomes 1 and 8. Amplification of transcription factors like PROX1 and NR5A2 was observed. Additionally, the transcriptomic profile of KRAS wild-type PDAC shared similarity with cholangiocarcinoma, so therapeutic approaches used in biliary tract cancers may also be taken into consideration in KRAS wild-type PDAC [53].

### 3.5. Amplification of KRAS

Amplification of KRAS is the fourth most common KRAS alteration. Its occurrence has been observed in esophageal, stomach, and serous ovarian cancer. Particularly aggressive phenotypes in PC are supported by the levels of mutant KRAS [54]. A case report by Pittella-Silva et al. documents a patient with advanced PDAC and multiple liver metastases, who was found to have a significantly amplified oncogenic KRAS G12D allele. The amplification of this allele was linked to rapid tumor development and a poorer response to chemotherapy [49]. Another study indicated that amplifications of KRAS G12C occurred as acquired resistance in patients with KRAS G12C-mutant PDAC who received treatment with adagrasib or sotorasib [55].

### 3.6. KRAS-Driven Initiation and Immune Modulation in PDAC

PDAC typically arises from two main precursor lesions: PanIN (85–90%) and intraductal papillary mucinous neoplasms (IPMNs, 10–15%). KRAS mutations appear early, even in low-grade PanIN, highlighting their key role in initiation, though additional mutations are needed for full progression [56].

PDAC is marked by a dense fibrotic stroma and an immunosuppressive TME, partly shaped by KRAS mutations. These mutations have cell-extrinsic effects, promoting immune evasion early in the disease. In mouse models, KRAS-mutant PanIN lesions show inflammation, COX2 overexpression, and early infiltration of immunosuppressive cells like T regulatory cells (Tregs), tumor-associated macrophages (TAMs), and myeloid-derived suppressor cells (MDSCs) [57].

KRAS mutations also drive the secretion of cytokines such as IL-6 and GM-CSF, activating pro-inflammatory pathways (e.g., JAK/STAT3) and recruiting MDSCs, further dampening anti-tumor immunity. Stromal cells also contribute by secreting factors that support invasion and metastasis. RAS signaling upregulates chemokines (e.g., CXCL8, CXCR2) and transcription factors like NF-κB, reinforcing inflammation and immune suppression. KRAS also boosts PD-L1 expression and promotes Treg formation through ERK activation and IL-10/TGF-β1 signaling. However, specific chemokine signatures (e.g., CCL4, CXCL9) can signal T cell infiltration and may predict the response to immune checkpoint therapies. This underscores the value of TME profiling in guiding combination treatments in PDAC [54].

## 4. Targeting KRAS Mutations

### 4.1. KRAS Inhibitors

As previously mentioned, KRAS mutations are found in most of PDACs. The discovery of irreversibly binding inhibitors against the KRAS G12C oncoprotein, which uses mutant cysteine for binding, is considered a significant advance. It increases the binding of the inhibitor by promoting a GDP-bound, inactive state [47,58]. Unfortunately, this action is not possible with non-G12C mutant Ras proteins. Tis is why there is still a need to develop drugs that can target other isoforms.

In 2021, based on the results from the CodeBreaK 100 trial (NCT03600883), the Food and Drug Administration (FDA) approved sotorasib (AMG-510) for the treatment of KRAS G12C-mutated locally advanced or metastatic non-small cell lung cancer (NSCLC) in patients previously treated with at least one systemic therapy [59]. The objective response rate (ORR) was 37.1%, with a median response duration of 11.1 months. The median progression-free survival (mPFS) was nearly 7 months [60]. Moreover, in the CodeBreaK trial, 38 patients with metastatic PC received sotorasib. Over 20% had ORR, with a median response duration of 5.7 months. The mPFS was 4.0 months, and overall survival (OS) was 6.9 months, better than the other second-line treatment regimen. A mainly low-grade toxicity profile was observed, where diarrhea and fatigue were the most frequently occurring symptoms [61]. This is why sotorasib may be a promising drug as a second-line treatment; however, more extensive studies should be conducted.

Another KRAS G12C inhibitor—adagrasib (MRTX849)—was approved by the FDA in 2022 for patients with NSCLC, following one prior systemic therapy [62]. Moreover, in 2024, it was approved by the FDA in combination with cetuximab for mutated locally advanced or metastatic CRC, after prior treatment [63]. It has favorable properties and dose-dependent pharmacokinetics. Despite a decrease in plasma levels, the long-term pharmacodynamic effect of MRTX849 is consistent with the relatively long half-life of the KRASG12C protein, approximately 24–48 h [64]. The phase I/Ib KRYSTAL trial (NCT03785249) assessed the efficacy of adagrasib in 25 patients with KRAS G12C-mutated solid tumors. After a median follow-up of 19.6 months, 8 of 15 patients with NSCLC achieved an established partial response. The median duration of response was 16.4 months, and the mPFS was 11.1 months. Also, one of two patients with colorectal cancer achieved a partial response with a duration of 4.2 months. Adverse effects (AEs) were primarily low-grade and gastrointestinal, including nausea, diarrhea, vomiting, and fatigue [65]. In the updated analysis, at the data cutoff of 10 September 2021, 42 patients were enrolled, and 12 had PDAC. In this group of cancers, mPFS was 6.6 months, and treatment was ongoing in 50% of patients [66]. The results from phase 2 (data cutoff 1 October 2022) showed that from the 64 patients enrolled, including 57 with measurable disease, the ORR was 35.1%, and the median duration of response was 5.3 months (*n* = 20; 95% CI 2.8–7.3). The mPFS was 7.4 months, and OS was 14 months. From the cohort, 21 patients with PDAC had a similar ORR, about 33%, but the mPFS and OS were lower, at 5.4 months and 8 months, respectively [67]. However, adagrasib was well tolerated and showed promising activity in pretreated patients. Currently, the phase III KRYSTAL-12 trial (NCT04685135) will assess the efficacy of adagrasib versus docetaxel in patients with advanced NSCLC and KRAS G12C mutation who have progressed during or after treatment with a platinum-based regimen and an immune checkpoint inhibitor [68].

KRAS G12C mutations rarely occur in PDAC, creating a need to discover inhibitors for other mutations. In recent years, the potent KRAS G12D inhibitor MRTX1133 has shown promising results in preclinical studies, both in vivo and in murine animal models [69,70]. It exhibits high selectivity for binding to the GDP-bound inactive KRAS G12D and inhibits the binding of the RAF-RAS binding domain peptide to the active form of KRAS G12D. Moreover, MRTX1133 has demonstrated significant tumor regression in immunocompetent models of KRAS G12D, leading to either complete or nearly complete remission. Regression of PanIN was also observed. However, there are challenges, like resistance to the drug or low oral bioavailability, that should be further discussed [70]. Currently, its activity is set to be assessed in a first-in-human phase 1 clinical trial, NCT05737706, involving patients with advanced solid tumors harboring a KRAS G12D mutation [71]. Another selective inhibitor, HRS-4642, showed a tolerable safety profile and antitumor activity in patients with solid tumors in the phase I NCT05533463 study. Treatment-related adverse events were observed in 33.3% of patients, but no one discontinued the treatment. However, only one of 18 patients enrolled had PC, so there is still a need for further investigation of KRAS G12D inhibitors in this type of cancer [72].

According to that another approach to inhibit the KRAS G12D mutation is by degrading KRAS using proteolysis targeting chimeras (PROTACs). These chemicals facilitate the spatial closeness of a target protein to an E3 ubiquitin ligase, subsequently tagging the KRAS protein with ubiquitin, which is recognized by proteasomes for degradation [73]. ASP3082 has shown preclinical efficacy in KRAS G12D-mutant NSCLC mouse models, and its effectiveness is under investigation in a phase I study, NCT05382559, with previously treated patients [74]. Preliminary results indicated an acceptable safety profile and promising antitumor activity. AEs were observed in 69.4% of patients (68/98). However, no grade 4 or 5 AEs existed. The most common AEs reported included fatigue, infusion-related reactions, pruritus, nausea, urticaria, increased aspartate aminotransferase (AST), alanine aminotransferase (ALT), and vomiting. The maximum tolerated dose was not reached (600 mg); however, at 300mg, the ORR was 33.3%, and the disease control rate (DCR) was 75%. Of all enrolled patients, 67 had PC, so this type of therapy could be an option in heavily pre-treated patients [75]. Other degraders, RP03707 and HDB-82, were recently discovered and showed excellent antiproliferative activity, with favorable pharmacokinetic and pharmacodynamic properties in mouse models [76,77]. The first one, unlike classical inhibitors, induces the proteasomal degradation of KRAS G12D by recruiting cereblon (CRBN) E3 ligase. It exhibited a stronger antiproliferative effect than MRTX1133 [76]. Further studies are required to assess the KRAS G12D degrader’s efficacy fully. There are also obstacles to PROTAC usage due to its high molecular weight and complex structure, which limit drug passage through the cell membrane. They are outside of Lipinski’s rule-of-5, which may result in poor bioavailability. The second problem is that non-covalent or irreversible PROTACs may exhibit numerous disadvantages, including diminished binding affinity and erratic off-target effects. On the other hand, reversible covalent PROTACs have higher potency and selectivity, as well as a longer duration of action. Even though this technology offers a wide range of potential clinical applications for undruggable targets, it must overcome key challenges related to bioavailability, tissue-selective E3 ligases, and rational linker optimization [78].

Unlike conventional PROTACs, Chemical knockdown with Affinity aNd Degradation DYnamics (CANDDY) technology was proposed as an alternative strategy for PROTACs, using ubiquitination-independent degradation by delivering the target protein directly to the proteasome via a modified degradation tag derived from proteasome inhibitors. Miyamoto-Sato et al. created TUS-007, which has both a KRAS-binding moiety and a CANDDY tag. It can induce the targeted degradation of both KRAS mutants G12D and G12V, but also wild-type KRAS, without difficulties in matching targets and specific ubiquitination-related proteins. Tumor suppression was observed in xenograft models of colon and pancreatic cells [79]. Further research is needed to evaluate its translational potential and therapeutic applicability.

Compared to KRAS G12C or KRAS G12D, the KRAS G12V mutation has a substantially reduced intrinsic GTP hydrolysis rate. Therefore, to minimize this oncogenic driver as much as possible, it is essential to target the KRAS G12V in its active state. Newly developed small-molecule inhibitors of the active form of KRAS G12V act by inducing the formation of a ternary complex with cyclophilin A (CypA) and active KRAS G12V, which leads to the destabilization of the interaction of KRAS with effectors like RAF. In human tumor xenograft models with mutated solid tumors, these inhibitors showed durable tumor regression and were well tolerated [80]. The consequence of this mechanism is inhibiting the RAS/MAPK pathway, which results in a strong antiproliferative effect and the induction of apoptosis in cancer cell lines with this mutation. Additionally, KRAS G12V degradation stimulates a potent antitumor immune program, potentially driven by activated CD8+ T cells. It leads to an increased expression of pathway genes involved in the inflammatory response and the IFN-γ and IFN-α response. The antitumor response to KRAS G12V degradation has been observed to depend on the presence of CD8+ T cells. This is further supported by the fact that KRAS G12V degradation causes an increase in the number of macrophages with the M2 phenotype, which is active in tumor promotion, and a specific number of macrophages with the M1 phenotype, which is antitumor [81]. Further investigation is crucial to determine the clinical feasibility, including immune-related toxicity and pharmacokinetics optimization [81].

In Zhang et al.’s study, covalent chemical ligands for the KRAS G12R mutation were discovered. They bind in the switch II pocket and irreversibly react with the mutant weakly nucleophilic arginine residue. Although they do not show activity in KRAS G12R mutant cells, this work provides an area where other improvements may be conducted [82].

In recent years, there has been enormous progress in creating druggable targets for KRAS-mutant PDAC; however, the clinical applicability remains limited. Two approved inhibitors, adagrasib and sotorasib, could be useful only in 1–3% of patients harboring the KRAS G12C mutation, and they showed modest clinical benefit in PDAC. Other selective inhibitors for G12D and G12V mutations are still under investigation to evaluate their effectiveness. There is also the problem of resistance and disease progression, which can be observed in patients [83].

### 4.2. Pan-RAS Inhibitors

Pan-RAS inhibitors target a broad spectrum of mutant RAS and wild-type RAS when overactive in a specific tumor context. As was mentioned before, PDAC is commonly heterogeneous, so selective KRAS inhibitors may be insufficient. Using Pan-RAS inhibitors is one of the promising options for a more complete suppression of RAS signaling by inhibiting all RAS isozymes regardless of mutational status [84].

Kim et al. described a non-covalent inhibitor, BI-2865, which blocked wild-type KRAS and a wide range of KRAS mutants, including common mutations found in PDAC, such as G12D, G12V, G13C, and G13D. It preferentially inhibited the inactive state of KRAS (KRAS(OFF)), while sparing HRAS and NRAS, which enhanced its selectivity and improved toxicity profile. However, in wild-type KRAS, the treatment led to increased activation of other RAS isoforms, limiting the drug’s effectiveness. In mouse xenograft models with various KRAS mutations (G12C, G12D, G12V, A146V), BI-2865 inhibited tumor growth without visible adverse effects on animal body weight [85].

ADT-007 is a first-in-class pan-RAS inhibitor that could address the complex landscape of RAS mutations and has better resistance-avoiding capabilities than other RAS inhibitors. It also binds nucleotide-free RAS to block the GTP activation of effector interactions and MAPK/AKT signaling in wild-type RAS cancer cells [86]. ADT-1004 (an oral prodrug of ADT-007) also showed strong antitumor activity in the PDAC model. Moreover, it had greater efficacy than sotorasib, adagrasib, and MRTX1133; however, it did not impact the growth of wild-type RAS PDAC cells [87].

The pan-RAS inhibitors mentioned bind to a pocket between switches I and II in the KRAS(OFF) state. Recently, efforts were made to create inhibitors that would bind to active-state KRAS [88]. For the KRAS G12D mutation, the RMC-9805 showed selective inhibition in KRAS G12D human cancer cell lines in vitro and tumor models in vivo [89]. Daraxonrasib (RMC-6236) is a nonsteroidal, noncovalent multi-selective inhibitor of the active form of RAS (RAS(ON)), both mutated and wild-type. Its action mechanism involves forming a tri-complex with CypA and the active form of RAS [88,90]. In vitro studies have shown its efficacy on various forms of RAS, and in animal models, tumor regression with various KRAS mutations (G12D, G12V, G12C) was dose-dependent. In the ongoing phase I/Ib clinical trial (NCT05379985), two patients, one with NSCLC and one with PDAC, showed an objective response [90]. The most common AEs were rash, diarrhea, nausea, vomiting, stomatitis, fatigue, paronychia, mucosal inflammation, decreased appetite, and peripheral edema. The ORR, PFS, and OS showed promising efficacy in previously treated patients with RAS-mutant PDAC. The mPFS was 8.1 months in patients with the KRAS G12X mutation and 7.6 months in those who broadly have RAS-mutant tumors [91]. Currently, not only is the III phase of NCT06625320 trial is ongoing, but several clinical trials are investigating the efficacy and safety of daraxonrasib and RMC-9805 (NCT06040541; NCT06162221; NCT06445062; NCT06128551) [92,93,94,95,96,97]. They are all presented in Table 1. In the phase I NCT06040541 study, RMC-9805 showed preliminary antitumor activity with early and deep reductions in KRAS G12D ctDNA in patients with KRAS G12D PDAC. No grade 4 or 5 AEs were reported. The most common grade 1 or 2 AEs were nausea, diarrhea, vomiting, and rash [98].

YL-17231 also functions as a pan-RAS inhibitor, and its safety, tolerance, pharmacokinetics, and maximum tolerance are currently being investigated in patients with advanced solid tumors harboring KRAS mutations in the phase I NCT06078800 study [99]. In a series of in vitro and in vivo pre-clinical models, it showed robust activity, and also in KRAS G12C drug-resistant cells. Moreover, the pharmacokinetics and oral bioavailability were satisfactory [100]. An overall summary of novel KRAS-inhibitors and PROTACs molecules is presented in Table 2.

### 4.3. RNA Interference (RNAi)

A post-transcriptional mechanism known as RNA interference (RNAi) promotes the cleavage of the appropriate mRNA to block the expression of a particular gene. RNAi consists of two major types of RNA molecules: siRNA and miRNA, which are short non-coding RNA strands [101]. Six RNAi-based drugs have been approved by the FDA: patisiran, givosiran, lumasiran, inclisiran, vutrisiran, and nedosiran, in 2018, 2019, 2020, 2021, 2022, and 2023, respectively [102]. Currently, numerous ongoing studies are demonstrating the potential of RNAi drugs in treating neurodegenerative and cardiovascular diseases, as well as their effectiveness against various viruses, including HIV and the hepatitis C virus. However, cancer remains a primary focus of RNAi-based treatment [103]. In PC cells, RNAi-mediated inhibition of mutant KRAS expression decreased tumorigenic growth, anchorage-independent growth, and proliferation [104].

#### 4.3.1. Small Interfering RNA

**Figure 3:**
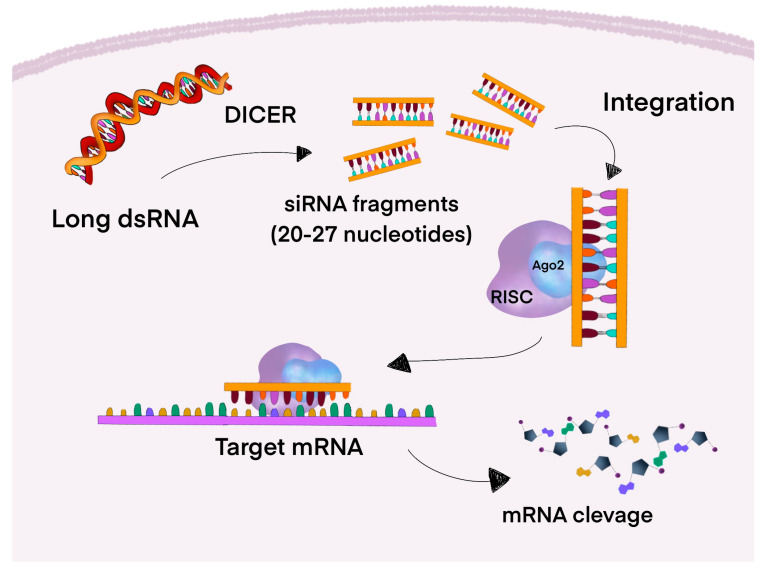
Mechanism of formation and action of siRNA. Dicer, by splitting dsRNAs, forms molecules with 20–27 base pairs in length called siRNAs. Created siRNAs are integrated into a complex structure made up of AGO2 and RISC. Next, the targeted mRNA is bound to the antisense strand of the siRNA, leading to its cleavage. This mechanism prevents the translation of the targeted mRNA into a functional protein [105]. Abbreviations: dsRNA—long double-strand RNA; Dicer—dsRNA-specific RNase; siRNA—small interfering RNA; AGO2—Argonaute 2; RISC—RNA-induced silencing complex.

Functioning through the RNAi pathway, small interfering RNA (siRNA) is a family of double-stranded non-coding RNA (ncRNA). As illustrated in Figure 3, siRNAs are molecules with lengths ranging from 20 to 27 base pairs, whose precursors, encoded in the genome, are long double-stranded RNAs (dsRNA). Dicer, a dsRNA-specific RNAse, cleaves long dsRNAs into siRNA fragments that contain 20 to 25 nucleotides with two nucleotides in the 3′ overhang and 5′ phosphate groups. Subsequently, the siRNA is integrated into a complex structure comprising the catalytic core of the Argonaute 2 (AGO2) protein and the RNA-induced silencing complex (RISC). The cleavage pathway is activated when a specific target mRNA, possessing a perfectly complementary sequence, binds to the antisense strand of the siRNA. By cleaving the target mRNA into small fragments, the AGO2 endoribonuclease prevents the mRNA from being translated into a functional protein. Synthetically produced siRNAs, after exogenously transferring into cells or organs, are quickly loaded into the RISC-AGO2 complex, resulting in the effective degradation and inhibition of the target mRNA [105].

A clinical trial was conducted in 2015 by Golan et al. using a siG12D-LODERTM (Local Drug Eluter). This miniature biodegradable polymeric matrix contains anti-KRAS G12D siRNA for non-operable Locally Advanced Pancreatic Cancer (LAPC) patients. The siG12D-LODERTM was implanted into a tumor and released the drug over four months. Fifteen patients were divided into three increasing dosage cohorts (0.025 mg, 0.75 mg, and 3.0 mg) and received a single dose of siG12D-LODERs. One of the enrolled patients did not receive chemotherapy, while fourteen received the concomitant standard of care (SOC). As a result, the median OS was 15.12 months, and the median time to metastasis (TTM) was 8.25 months. Additionally, the 18-month OS was 38.5%, and the TTM was 15.4%. There was no significant difference in TTM among these three dose cohorts. Most AEs were graded as 1–2, with the most frequently observed being diarrhea, abdominal pain, nausea, and fatigue. These findings suggest that the siG12D-LODERTM implant, in combination with chemotherapy, is a safe and potentially effective treatment for patients with LAPC, showing promise for siRNA-based drugs [106]. Although siRNA-loaded implants like siG12D-LODER show promise in terms of safety and effectiveness, greater clinical use necessitates resolving issues including implant scalability, intratumoral delivery uniformity, and sustained release control in various tumor contexts [107].

Another approach targeting KRAS G12D is being investigated in a phase I NCT03608631 study where mesenchymal stromal cells-derived exosomes with siRNA (iExosomes) are administered intravenously to patients with metastatic PDAC. The primary objectives are to identify the maximum tolerated dose and dose-limiting toxicities [108].

In the study by Anthiya et al., functionalized lipid nanoparticles (LNPs) were designed to deliver siRNA intended to silence the *KRAS* gene. A potent anti-KRAS siRNA covering multiple forms of KRAS (pan-KRAS) was identified. The siRNA was next loaded into LNPs, both tLyp-1 peptide-targeted and non-targeted variants. The biodistribution and therapeutic efficacy of the siRNA-loaded LNPs were assessed using a murine model with subcutaneous CFPAC-1 (human pancreatic cell line harboring a KRAS G12V mutation) PC xenografts. The targeted LNPs exhibited enhanced tumor accumulation and significantly decreased tumor growth when paired with gemcitabine, making it a practical and encouraging approach to creating a cancer therapy [109]. Nonetheless, there are still possible translational obstacles. LNP-based siRNA treatments may encounter difficulties such as lipid component immunogenicity, off-target effects, and dose-dependent toxicity in humans, despite the encouraging tumor growth and effectiveness in mouse models [110]. Furthermore, before translation to patients is possible, scalability concerns related to the stability and large-scale production of siRNA-LNP formulations for clinical use must be resolved [111].

Another interesting study was performed by Küçükekmekci et al., aiming to silence the *KRAS* gene in PC using siRNA and gold nanoparticles (AuNP). The cell line, CAPAN-1, used in this study was exposed to 25 nM siRNA and AuNP at concentrations of 0.5 mg/mL, 0.25 mg/mL, and 0.125 mg/mL. The results of real-time PCR analysis showed that this complex effectively silenced the *KRAS* gene. The WST-1 assay showed minimal toxicity. Moreover, the xCELLigence real-time cell analysis revealed a significant reduction in cell proliferation in cells treated with this complex compared to controls. This study’s findings imply that the AuNP/siRNA complex can successfully silence the targeted gene [112]. However, more research is required to determine the in vivo biodistribution, potential immunogenicity, and long-term safety of gold nanoparticles, particularly regarding organ accumulation and clearance mechanisms, which are crucial for clinical translation, even though the AuNP/siRNA complex showed promising gene silencing and low in vitro toxicity [113].

A study performed by Strand et al. used peptide-based, oligonucleotide-condensing, endosomolytic NPs for siRNA delivery. For this study, murine pancreatic cells (KPC-1) were used. As a result, a 69% decrease in KRAS mRNA expression and a 45% decrease in cell viability were observed. Moreover, fluorescently tagged NPs administered intravenously resulted in a significant accumulation in murine models with subcutaneous KPC-1 tumors, suggesting efficient tumor targeting. NPs were also able to penetrate the dense stromal barriers characteristic of PDACs, showing the high therapeutic potential of this method [114]. Nevertheless, there are several challenges in the clinical translation of peptide-based nanoparticles. Effective endosomal escape is still difficult to achieve, which frequently restricts the therapeutic effect and cytoplasmic siRNA delivery [115]. Furthermore, it is physically challenging to produce such nanocarriers at a wide scale with consistent quality, and peptide sequences may activate innate immune responses [116].

A recently published study by Shirvan et al. used SIL-204 to treat localized cancer with KRAS G12x and G13D mutations. SIL-204 contains siRNA, designed to silence G12x and G13D KRAS mutations, encapsulated within biodegradable polylactic-co-glycolic-acid (PLGA) microparticles. SiRNA is released after intratumoral injections. In vivo studies showed decreased tumor growth; enhanced therapeutic outcomes were observed compared to non-encapsulated siRNA treatment because the extended-release formulation guaranteed continued siRNA administration [117]. Despite these benefits, the use of PGLA-based systems is restricted by the requirement for intratumoral injections, which is difficult in cases of metastatic illness and may cause inflammatory reactions during breakdown. Additionally, scaling difficulties are raised by problems including batch repeatability, loading efficiency, and particle size variability [118].

A recently published article by Stanland et al. introduces EFTX-G12V, an RNAi-based therapeutic that uses fully chemically modified small interfering siRNA, which inhibits KRAS G12V expression at mRNA and protein levels. A non-mitogenic epidermal growth factor receptor (EGFR) ligand is bound to the siRNA, improving tumor-specific delivery, especially to tumors with different EGFR expression levels. EFTX-G12V showed strong anti-tumor activity in lung and colon cancer xenograft models without having any off-target effects on wild-type KRAS in physiological tissues such as the kidney, skin, and bladder. Compared to pan-KRAS siRNA methods, EFTX-G12V showed a more effective inhibition of tumor angiogenesis [119]. Despite EFTX-G12V’s improved off-target toxicity and mutation specificity, difficulties still occur. Depending on their backbone chemistry and mode of delivery, chemically modified siRNAs can still trigger immunological responses [120]. Furthermore, different tumor cells’ EGFR expression can result in less effective targeting, and the high cost of producing customized siRNAs prevents widespread clinical use [121].

siRNA-based therapies have emerged in recent years as a promising strategy for cancer treatment. Therapies targeting the KRAS mutation aim to silence mutant KRAS mRNA, preventing its translation and thus inhibiting tumor progression. The siG12D-LODER^TM^, a biodegradable implant loaded with KRAS G12D-targeting siRNA, is potentially a safe and effective treatment for LAPC. Another potential way of delivering siRNA is using LNPs that carry pan-KRAS siRNA, which showed improved tumor targeting and enhanced therapeutic effects when combined with gemcitabine. PLGA microparticles, designed by Shirvan et al., deliver siRNA targeting KRAS G12x/G13D mutations, offering sustained release and enhanced antitumor activity. EFTX-G12V, a chemically modified siRNA conjugated to an EGFR ligand, introduced by Stanland et al., showed a potent inhibition of KRAS G12V in lung cancer models without affecting wild-type KRAS in physiological tissues. Summarizing the result, there has been enormous progress in the research and development of siRNA-based therapies. Targeted siRNAs, such as EFTX-G12V, show potential mutation-specific treatment, whereas a nanoparticle-based delivery system will enhance delivery efficiency. Newly developed release formulations offer prolonged exposure, and combination therapies, such as siRNA with gemcitabine, show synergistic benefits [106,109,112,114,117,119]. Despite the lack of approved siRNA-based therapy for KRAS-mutated PDAC, new research shows promising perspectives for the future.

#### 4.3.2. MicroRNA

MicroRNAs (miRNAs) are 19–24-nucleotide non-coding RNAs [122]. MiRNA can be classified into two categories: oncogenic, which inhibits the expression of tumor suppressor genes, and tumor-suppressing, which inhibits the expression of oncogenes. When oncogenic and tumor suppressor miRNAs are dysregulated, the dynamic balance between oncogenes and tumor suppressor genes is broken, leading to the growth and development of tumors [123]. RISC contains activated miRNA, and this complex will attach to the targeted mRNA, usually in a conserved location known as the seed region [124]. By base-pairing to complementary sites in the 3′-untranslated region (UTR) of target mRNAs, miRNAs control gene expression by causing translational repression or mRNA destruction. It has been discovered that in PDAC, miR-217, which is responsible for cell proliferation, is significantly downregulated. When miR-217 is overexpressed, the KRAS levels are decreased, and cell proliferation is inhibited. Because miRNA suppresses KRAS, its restoration has potential as a therapeutic factor for treating PDAC [122].

A study performed by Zhao et al. investigates the role of microRNA-217 (miR-217), mentioned above, in regulating the KRAS and its potential as a tumor suppressor in PDAC. They discovered that the expression of miR-217 was reduced in 76.2% of PDAC compared to normal pancreatic tissues. KRAS was identified as a potential direct target of miR-217. Moreover, dual-luciferase reporter assays confirmed that it binds to the 3′-UTR of KRAS mRNA, resulting in decreased levels of KRAS protein and reduced phosphorylation of downstream protein kinase B, also known as AKT. In conclusion, miR-217 is a tumor suppressor in PDAC [125]. Unfortunately, there are significant obstacles to miR-217 translational application. First, the dense desmoplastic stroma that restricts medication penetration makes it difficult to deliver drugs to pancreatic tumors effectively [126]. Second, because of their capacity to control several genes and the possible cytotoxicity of delivery carriers, miRNAs such as miR-217 may have toxicity or off-target effects [127].

Yu et al. explored the role of microRNA-96 (miR-96) in regulating KRAS and its potential as a tumor suppressor in PC. Using bioinformatics analysis, a conserved binding site for miR-96 was found in the 3′-UTR of KRAS mRNA. Luciferase reporter assays confirmed the direct binding of miR-96 to this site and indicated the direct interaction and suppression of KRAS expression. Moreover, quantitative real-time PCR showed that miR-96 is significantly downregulated in PC compared to normal pancreatic tissue, correlating with elevated KRAS expression. Overexpression of miR-96 reduced KRAS protein levels, AKT inhibition, and apoptosis induction. In in vivo mouse models, tumors generated from cells expressing miR-96 showed slower growth rates and reduced tumor size [128]. However, the strong stromal barrier and limited vascularization in PDAC limit the accumulation of therapeutic RNA molecules at the tumor site, making efficient and tumor-specific delivery challenging [129]. Moreover, before clinical translation is possible, it is necessary to address the potential for off-target effects or immune responses from miRNAs like miR-96, as well as the potential for cytotoxicity from certain delivery systems [130].

Another study investigating miRNA as a tumor suppressor was performed by Mokhlis et al., investigating miR-873 in PDAC and triple-negative breast cancer (TNBC). Comparable results were observed in the studies mentioned above. Expression of this miRNA was reduced in PDAC and TNBC tissues compared to normal tissues, which was associated with shorter OS in patients with these cancers. Bioinformatics analyses identified KRAS mRNA as a direct target of miR-873, and luciferase reporter assays verified that miR-873 binds to the 3′-UTR of KRAS mRNA. Using nanoparticles, miR-873 was delivered to mouse models of PDAC and TNBC, where decreased tumor growth and increased tumor cell apoptosis were observed [131]. Although preclinical models have demonstrated the potential of nanoparticle-based delivery, concerns like effective and targeted distribution, possible toxicity, immunological reactions, and scalability must be carefully considered to guarantee clinical viability [132].

Garibaldi-Ríos et al. recently published a study exploring the role of miRNAs in regulating KRAS in PC through computational analyses. Here, 210 dysregulated miRNAs were found in PC tissues, of which 116 were overexpressed and 94 were underexpressed. It was discovered that 16 miRNAs may control the expression of KRAS, and 9 of them show correlations with PC’s clinical features. Increased mortality was associated with overexpression of hsa-miR-30a-5p, and patient survival rates were associated with three of them. Moreover, 10 miRNAs were predicted to have a regulatory impact on KRAS [133]. Before clinical implementation, additional in vivo validation and functional research are necessary, even if these computational predictions provide insightful information about regulatory networks. Furthermore, because miRNA regulation is pleiotropic, applying such multi-target miRNA methods to therapy presents issues with dose control, delivery specificity, and off-target interactions [134].

Depending on their targets, miRNAs can function as either oncogenes or tumor suppressors. In PDAC, dysregulation of the balance between these roles contributes to tumor progression. Studies have identified specific mRNAs that function as tumor suppressors by targeting KRAS. MiR-217 has been found to be downregulated in PDAC, while its overexpression inhibits cell proliferation and tumor growth. Another miRNA downregulated in PDAC is miR-96, whose overexpression resulted in smaller tumor growth in vivo. The delivery of another miRNA, miR-873, via nanoparticles inhibited tumor growth in mouse models. Overall, 210 dysregulated miRNAs have been found in PDAC tissues, including sixteen that regulate KRAS. Nine studies correlated with patient survival and mortality [125,128,131,133]. Tumor-suppressive miRNAs can serve as a therapeutic strategy to downregulate KRAS and inhibit tumor progression. In contrast, miRNAs linked to prognosis and survival can be utilized as biomarkers for treatment selection. For example, such miRNAs can aid in early diagnosis. Summarizing the results of the studies mentioned above, the future of miRNA-mediated KRAS inhibition in PDAC is highly promising. Moreover, miRNAs could become a powerful tool in the fight against PDAC.

#### 4.3.3. Challenges of RNAi-Based Therapies

Despite the creation of new RNA-based therapeutics, effective delivery of these drugs into the cancer cells remains a major challenge. Macropinocytosis is a mechanism of delivering nutrients to cancer cells within the nutrient-poor tissues. For this reason, it is widely used in delivering RNAi-based drugs. However, when an RNAi drug enters the cell, it is transported to early endosomes. Next, they are transported to late endosomes and, ultimately, lysosomes. Lysosomes are rich in nucleases, enzymes that degrade RNA. A significant obstacle is the creation of delivery systems that can target tumor cells specifically, evade immune identification, and avoid endosomes. Although aptamer-conjugated platforms, lipid carriers, and nanoparticles are being researched, none of them have consistently worked in PDAC because of the tumor’s stroma complexity and inadequate vascularization. Moreover, the lack of specific inhibitors, the distinct cell types with varying rates, high sensitivity to external stimuli, and other factors have made it difficult for investigations to pinpoint the precise mechanism of macropinocytic distribution [101,135]. Another challenge concerns the TME. Most of the tumor mass in PDAC is made up of a desmoplastic stroma. This fibrotic barrier and aberrant vasculature hinder the disruption and penetration of therapeutic drugs within the tumor tissue. Additionally, the effectiveness of the treatment is even more difficult due to the immunosuppressive character of the TME [101,136]. Resistance mechanisms also limit the success of RNAi therapies. Dilly et al., in a study published in 2024, identified both genetic and non-genetic adaptations, including the activation of PI3K-AKT-mTOR signaling pathways and the induction of EMT, as key contributors to therapeutic resistance in PDAC [55]. These results underline the significant intratumoral heterogeneity of PDAC, where various cellular subpopulations may rely on KRAS signaling in different ways, hence restricting the effectiveness of targeted RNA interference strategies [137]. Mutations of KRAS in PDAC are diverse, with G12D and G12V being the most prevalent, making the creation of RNAi treatment more difficult. Structural variations among these subtypes necessitate RNAi constructs with almost perfect complementarity. For instance, when used on tumors with G12V or G12R mutations, therapies that target G12D may be less effective or have a higher risk of off-target effects [101,138,139].

## 5. Resistance and Targeting Ras Effector Pathways

The MAPK and PI3K pathways are key in mediating the proliferation, survival, and metabolic effects of KRAS. Eventually, the dimerization of BRAF with CRAF and activation of CRAF can lead to activation of this pathway, although BRAF was inhibited. Moreover, inhibitors targeting the MAPK/ERK pathway did not show promising results in PC treatment [140,141,142]. Inhibition of KRAS effector pathways is hard to achieve because many metabolic pathways are connected to this oncogene [47]. Additionally, based on genetic evidence, complete removal of MEK and ERK expression efficiently prevents RAS-driven tumors; however, the toxicity is unacceptable, so there is a need for creating inducible knock-in strains that will substitute kinase-dead isoforms for the wild-type proteins [143].

During treatment with KRAS G12C inhibitors, increasing resistance is observed, which creates a major therapeutic problem and the need to change therapy. Currently, the mechanisms of resistance are not well understood. Among patients with KRAS G12C-mutant cancers treated with adagrasib monotherapy in the KRYSTAL-1 trial, Awad et al. (2021) showed that patients acquired novel secondary KRAS mutations within the adagrasib-binding pocket. Several gained switch II pocket mutations like Y96C, H95Q, R68S, H95D, and H95R disrupt the drug’s noncovalent binding interactions. Moreover, the other activating mutations in KRAS, like G12D, G12V, G13D, G12R, and Q61H, were observed. Genetic mutations harboring a binding pocket in the KRAS protein were not the only problem. Alterations in other receptor tyrosine kinase (RTK)–RAS–MAPK pathways have also been detected, including gene mutations in *NRAS*, *BRAF*, *MAP2K1/MEK1*, *EGFR*, and oncogenes like *CCDC6-RET* and *EML4-ALK.* Lastly, in two patients, *MET* amplification was the only potential genomic mechanism of adagrasib resistance [83]. According to the progression problem, in the KRYSTAL-2 trial (NCT04330664), the combination of MRTX849 and TNO155 is being tested in patients with advanced solid tumors with KRAS G12C mutations [144]. TNO155 is an allosteric inhibitor of wild-type SHP2, which in the CTNO155 × 2101 (NCT03114319) trial showed favorable pharmacokinetic properties and promising early safety and tolerability data [145]. Normally, SHP2, encoded by the *PTPN11* gene, dephosphorylates inhibitory sites in tyrosine kinases, resulting in downstream RAS/MAPK pathways, leading to the activation of the downstream RAS/MAPK pathway and promoting cell proliferation and survival. It also plays an important role in the programmed cell death pathway (PD-1/PD-L1) by supporting cancer tumor evasion [146]. Therefore, combining adagrasib and SHP2 inhibitors would prevent KRAS from reloading with GTP, resulting in more substantial and sustained tumor growth inhibition [144,146,147].

In KRAS-mutant lung cancers and colon cancer, MEK inhibition can activate the MAP2K4-JNK-JUN feedback loop, leading to activation, especially of ERBB3 and FGFR1, which can reactivate MEK and AKT signaling [148,149]. In PDAC, integrin-linked kinase (ILK)-mediated phosphorylation of the mTORC2 component Rictor and AKT can lead to resistance to KRAS or MEK inhibition. This is why the combination of either KRAS G12C or MEK inhibitors (MEKi) and mTORC1/2 inhibitors was evaluated in the Brown et al. study. mTORC2 activates AKT kinase downstream of the mTORC1 complex, leading to protein translation. In the study, the combination induced suppression and regression of the tumor, compared to single-agent treatments. Moreover, mice tolerated it with no gross tissue toxicities [150]. However, the combination therapy should have a good therapeutic window with distinct genotype-specific toxicity in KRAS mutant PDAC cells compared to KRAS wild-type cells in normal tissues [47].

The RAS-RAF-MEK-ERK pathway is strictly connected to the SOS1 protein. This is why MRTX0902, a selective SOS1 inhibitor which disrupts SOS1:KRAS protein–protein interaction, showed promising results in preclinical models, and its efficacy in combination with adagrasib is being investigated in a phase 1/2 trial (NCT05578092) in solid tumors harboring mutations in the KRAS pathway [151]. In Hillig et al.’s study, the inhibitor of SOS1—BAY-293—caused a reduction in pERK activity by about 50% by inhibiting the GEFs. Additionally, in combination with ARS-853 (covalent KRAS G12C inhibitor), it showed synergistic antiproliferative activity in the KRAS G12C-mutated cell line [152]. Hofmann et al. showed that BI-3406, in combination with MEKi, inhibits cellular proliferation in KRAS-driven cancers [153]. One of the SOS1 inhibitors—BI-1701963, closely related to BI-3406—has reached clinical trials of KRAS-mutated solid tumors alone or combined with a MEKi—trametinib (NCT04111458) [43,154]. In preliminary results from the monotherapy, it was well tolerated and showed signs of signaling modulation [155]. Norgard et al. studied SOS1 inhibitors (SOS1i) combined with trametinib, which controlled tumor growth in Kras-p53 mutant PDAC and rewired intercellular communication within the TME. Although the combination may enhance CD8+ T cell infiltration in specific contexts, it simultaneously induces strong immunosuppressive mechanisms by recruiting and polarizing M2 macrophages, increasing the number and activity of CAFs, and impairing dendritic cell maturation. These changes make tumors more susceptible to immunotherapy combinations like SOS1i + MEKi with anti-CD40, anti-PD-1, and anti-CTLA-4 agonists, leading to tumor clearance and durable immune memory [156].

## 6. Immunotherapy in Pancreatic Cancer

The treatment of PDAC is associated with an insufficient response. The TME has a hand in this. CAFs secrete chemokines such as granulocyte colony-stimulating factor (G-CSF), granulocyte-macrophage colony-stimulating factor (GM-CSF), CXCL2/5, and CXCL12. Therefore, the suppressive immune cells, including MDSCs, M2-like tumor-associated macrophages, N2 neutrophils, regulatory T cells, regulatory B cells, mast cells, and Th2 cells, flow to the tumor, resulting in the exhaustion of effector T cells and activation of CAFs. Then, CAFs promote desmoplasia in tumor tissue and cooperate with mast cells to encourage the growth of cancer cells [157]. The immune checkpoint molecules, such as PD-L1 and T cell immunoglobulins and immunoreceptor tyrosine-based inhibitory motif domain (TIGIT), are upregulated in the TME, whereas MHC-I is downregulated [157,158].

Anti-CTLA-4 and anti-PD-1/PD-L1 immune checkpoint inhibitors (ICIs)—durvalumab, tremelimumab, ipilimumab—have been used as targeted therapy due to the upregulation of checkpoint molecules, but the findings have been unsuccessful [159,160,161]. Research on MRTX1133 has shown changes in the TME by remodeling the CAFs and M1-TAMs, and increasing the amount of CD8+ T cell lymphocytes [43]. Combining MRTX1133 with immune checkpoint blockades such as anti-PD-1 or anti-CTLA-4 antibodies led to enhanced antitumor immunity, tumor eradication, and prolonged OS in preclinical models [162].

Ghukasyan et al. showed that stimulators of interferon gene (STING) agonists like diABZI in combination with MEKi can enhance the ability to induce type I IFN-dependent cell death [163]. Unlike other tumors, there is a high amount of STING in PDAC, which relates to alterations in tumor immunity [164,165]. MEK inhibition results in a greater NFκB activation in response to STING stimulation by causing the inhibition of NFκB stimulation. Synergistic effects can also increase the levels of poly(ADP-ribose) polymerase (PARP), and caspase 3 and 9, which increase the apoptosis of PDAC [163]. In addition, a recent study conducted by Jiang et al. examined the efficacy of triple therapy: autophagy co-inhibition (mefloquine) with MEK blockade (cobimetinib) combined with CD40 agonism (aCD40). It activated the STING/type I interferon pathway in PDAC cells, which in turn switched TAMs into a tumor-suppressive M1-like phenotype and achieved cytotoxic T cell activation in a mouse model [166].

A recent study on mouse models focused on cytokine-high CAF-derived cytokine IL-33 expression. It showed that epithelial KRAS-dependent stromal IL33 expression affected the pancreatic TME, as the loss of this cytokine increased CD8+ T cell infiltration and activation and, ultimately, reduced tumor growth, making IL33 another therapeutic target option [167].

## 7. Cancer Cell Metabolism

Cancer metabolism is key to survival, and in PDAC, KRAS mutations drive a distinct metabolic program centered on aerobic glycolysis. This metabolic shift supports an increased production of glucose, glutamine, and fatty acids, fueling growth and proliferation. These adaptations are often mutation-specific, with different KRAS variants influencing metabolism uniquely.

Metabolic changes in KRAS mutation towards anabolic pathways are crucial for cancer cell proliferation in PDAC. The early adaptive mechanism to the challenging microenvironment characterized by hypoxia and nutrient deprivation is known as the “metabolic switch” [168]. The aerobic glycolysis process is elevated (“Warburg effect”), amino acid metabolism undergoes reprogramming, and fatty acid and nucleotide biosynthesis are altered [54,168,169]. Furthermore, KRAS stimulates scavenging pathways such as macropinocytosis and autophagy, feeding the tricarboxylic acid cycle and sustaining growth [54,170], which has been explored in clinical trials as a target for autophagy inhibitors [171,172,173,174,175]. Autophagy also plays a crucial role in resistance against anti-cancer therapy and could potentiate chemotherapy when using inhibitors such as ATG4B inhibitor UAMC-2526 in the study by Takhsha et al. on mouse models [176]. Autophagy is a promising target in numerous clinical trials that combine hydroxychloroquine (HCQ) and MEK/ERK inhibitors to act synergistically, tested in both resectable and nonresectable or metastatic patients [171,177,178,179]. Macropinocytosis could also play a role in targeting KRAS-mutated cancers and has shown mechanistic differences in macropinocytosis in allele KRAS G12R, where impaired activation of the effector p110alpha PI3K occurs, exposing potential therapeutic vulnerabilities [180]. A study conducted by Chen et al. described the antitumor effect of ubiquitin-like protein 4A (UBL4A) on autophagy-related proliferation and metastasis in PDAC by directly targeting lysosome-associated membrane protein-1. It concluded that UBL4A might be a promising target for the treatment and prognostication of PDAC [181].

It is important to mention that the tissue of origin dictates the metabolic phenotype in KRAS-activated tumors, such as in PDAC and NSCLC. The same initiating events lead to different utilizations of branched-chain amino acids (BCAAs) [182]. NSCLC tumors incorporate free BCAAs as a protein source of nitrogen, while PDAC tumors exhibit lower BCAA utilization. Consequently, different enzymes are also required in KRAS-mutated tumors of different origins [182,183].

Santana-Codina et al. described how dependence on oncogenic KRAS correlates with specific metabolic profiles involving the maintenance of nucleotide pools as key mediators of KRAS dependence. As a result, they state that non-oxidative pentose phosphate pathway (PPP) or pyrimidine biosynthesis antagonization inhibits the growth of KRAS-resistant cells, and targeting nucleotide metabolism can overcome resistance to KRAS/MEK inhibition [184].

AVENGER 500 (NCT03504423), a phase III clinical trial, tested devimistat (CPI-613^®^), a novel lipoate analog that inhibits the tricarboxylic acid cycle at two key carbon entry points, in combination with modified FOLFIRINOX compared to FOLFIRINOX alone for patients with previously untreated metastatic PDAC [185]. Unfortunately, the results of this trial indicated that devimistat combined with modified mFOLFIRINOX did not enhance long- or short-term outcomes for metastatic PDAC patients compared to standard FOLFIRINOX, and there was no new toxicity associated with the addition of devimistat [186].

Further research into the metabolic properties of PDAC and its relationship with its TME may result in the development of novel therapeutic strategies. This research should focus on combination therapies, where metabolic pathway inhibitors are used in conjunction with various targets.

## 8. Future Management of KRAS-Mutated PDAC

This review highlights the urgent need to develop effective therapies for KRAS-mutated PC. Developing selective inhibitors for G12C, G12D, and G12V mutations, along with new degradation strategies like PROTACs and CANDDY, may be a promising path forward. However, there are challenges related to pharmacokinetics, bioavailability, molecular weight, and resistance mechanisms that require further investigation. Currently, only therapies for G12C mutations are approved; however, they rarely occur in PDAC. The ongoing RASolute trial could be a breakthrough in PDAC treatment [97].

Due to frequent resistance development, combination therapy with SHP2 inhibitors, MEKi, or mTOR inhibitors may enhance treatment response. Additionally, targeting and modulating the TME through IL-33 inhibition, STING pathway activation, or anti-CD40, anti-PD-1, and anti-CTLA-4 agonism may improve immune cell infiltration, resulting in better outcomes response.

**Figure 4:**
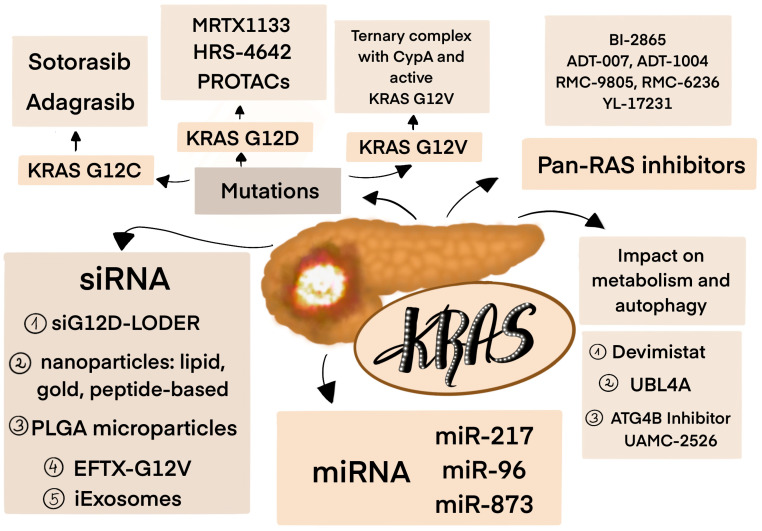
Novel treatment strategies for KRAS-mutated PDAC. Specific mutation-targeted strategies are currently being investigated, including selective inhibitors for KRAS G12C (sotorasib, adagrasib), KRAS G12D (MRTX1133, HRS-4642, PROTACs) and KRAS G12V (ternary complex formation with cyclophilin A, KRAS G12V and KRAS-inhibitor). Pan-RAS inhibitors (e.g., BI-2865, ADT-007, RMC-6236, YL-17231) target a broader range of KRAS isoforms, which may be more effective in heterogenous PDAC. Additional approaches involve RNA interference (siRNA therapies such as siG12D-LODER, nanoparticle-based delivery systems, PLGA microparticles, EFTX-G12V, and iExosomes), miRNAs (miR-217, miR-96, miR-873), and modulation of metabolic and autophagy pathways (e.g., devimistat, UBL4A, UAMC-2526). Based on [61,66,67,70,74,80,85,86,89,90,106,108,109,112,114,117,119,125,128,131,176,181,185]. Abbreviations: CypA—Cyclophilin A; PLGA—Poly(lactic-co-glycolic acid); siRNA—Small interfering RNA; miRNA—MicroRNA; KRAS—Kirsten rat sarcoma viral oncogene homolog; PDAC—Pancreatic ductal adenocarcinoma.

There is also significant progress in RNA-based therapies; however, their success in treatment is limited due to delivery barriers or increased immunogenicity. Future approaches should focus on developing improved transport mechanisms, such as exosome-based systems, which are currently under development investigation. Figure 4 summarizes novel treatment strategies for KRAS-mutated PDAC.

## 9. Conclusions

KRAS remains one of the defining and most prevalent mutations in PDAC. For many years, it was viewed as an undruggable oncogene. However, recent advances in research and clinical trials have led to the development of mutation-specific and pan-RAS inhibitors, which may be key to improving treatment. Despite this progress, challenges such as treatment resistance and the complex interactions between tumor cells, the stroma, and the immune system highlight the need for multimodal therapeutic strategies. Therefore, a deeper understanding of the molecular and metabolic landscape of KRAS-mutant PC is essential for developing more effective treatments. Recent years have seen significant milestones in cancer treatment improvement. A growing understanding of these mechanisms provides real hope for more effective therapies. SiRNA-based therapies have emerged as a promising cancer treatment strategy, targeting KRAS mutations to silence mutant KRAS mRNA, preventing its translation and inhibiting tumor progression. In contrast, miRNAs can serve as biomarkers linked to prognosis and survival. Additionally, combining KRAS-targeted therapies with immunotherapy, metabolic inhibitors, or RNA interference shows promise for enhancing therapeutic outcomes. Future clinical trials will be critical in assessing the efficacy and translational potential of these combination approaches.

## Figures and Tables

**Table 1:** Clinical trials evaluating the efficacy and safety of daraxonrasib and RMC-9805 [92,93,94,95,96,97].

Trial	Phase	Enrollment	Patients	Summary
NCT05379985	I	614	Patients with advanced solid tumors (NSCLC, CRC, PDAC) harboring specific RAS mutations	To evaluate safety, tolerability, PK, and clinical activity of escalating doses of RMC-6236. To determine the MTD and/or recommended phase 2 dose.
NCT06040541	I	444	Patients with KRAS G12D-mutant solid tumors	To evaluate the safety, tolerability, PK, and preliminary clinical activity of RMC-9805. Two arms: RMC-9805 monotherapy arm and RMC-9805 plus RMC-6236 combination arm.
NCT06162221	I II	484	Patients with RAS-mutated solid tumors with a focus on NSCLC	To evaluate the safety, tolerability, PK, and preliminary antitumor activity of novel RAS(ON) inhibitors combined with SOC or with each other: RMC-6291 +/− RMC-6236 + SOC RMC-6236 + SOC RMC-9805 +/− RMC-6236 + SOC.
NCT06445062	I II	1130	Patients with RAS-mutated solid tumors with a focus on GI cancers (PDAC, CRC)	To evaluate the safety, tolerability, PK, and preliminary antitumor activity of novel RAS(ON) inhibitors combined with SOC or with each other: RMC-6236 + 5-fluorouracil-based regimen RMC-6236 + cetuximab with or without mFOLFOX6 RMC-6236 + gemcitabine + nab-paclitaxel RMC-9805 with or without RMC-6236 + 5-fluorouracil-based regimens RMC-9805 with or without RMC-6236 + cetuximab with or without mFOLFOX6 RMC-9805 with or without RMC-6236 + gemcitabine + nab-paclitaxel.
NCT06128551	I	210	Patients with KRAS G12C-mutated solid tumors (CRC, PDAC, NSCLC)	To evaluate safety, tolerability, and PK profiles of RMC-6291 and RMC-6236.
NCT06625320 RASolute 302	III	460	Patients with metastatic PDAC who were previously treated with one prior line of therapy with a 5-FU-based or gemcitabine-based regimen	To evaluate the safety and efficacy of an RMC-6236 inhibitor compared to SOC treatment.

Abbreviations: PK—Pharmacokinetics; SOC—Standard(s) of Care; 5-FU—5-fluorouracil; NSCLC—Non-Small Cell Lung Cancer; PDAC—Pancreatic Ductal Adenocarcinoma; CRC—Colorectal Cancer; MTD—Maximum Tolerated Dose.

**Table 2:** Overview of KRAS Inhibitors in KRAS-mutated PDAC [61,67,68,71,74,75,78,85,86,87,90,91,92,93,94,95,96,97,99,100].

Inhibitor	Target Mutation	Phase	NCT Number(s)	Efficacy (ORR/mPFS/OS)	Pros	Cons
Sotorasib	KRAS G12C	II	NCT03600883	20%/4.0 months/6.9 months	FDA-approved for NSCLC, promising in PDAC	Limited to G12C (~1–3% in PDAC)
Adagrasib	KRAS G12C	II	1. NCT03785249, 2. NCT04685135	1. 33%/5.4 months/8.0 months 2. -	Promising second-line treatment; in combination with cetuximab	Limited to G12C (~1–3% in PDAC) Lower OS and mPFS results in PDAC group
MRTX1133	KRAS G12D	I	NCT05737706	-	Strong preclinical efficacy High selectivity;	Resistance and low oral bioavailability
ASP3082	KRAS G12D	I	NCT05382559	33.3% at 300 mg (early phase)	first-in-class PROTAC; safe profile	High MW limits cell penetration
RMC-9805	KRAS G12D	I	NCT06040541	Preliminary ctDNA reduction	Safety, with no grade 4 and 5 TRAEs	Early data only, need for further investigation
RMC-6236	Pan-RAS	I I, II I, II I III	NCT05379985, NCT06162221, NCT06445062, NCT06128551, RASolute302 (NCT05379985)	NCT05379985 mPFS 8.1 months for KRAS G12X mutation 7.6 months for broadly RAS-mutant tumors	Pan-RAS(ON) inhibitor; promising efficacy and safety profile	Still in trials, tolerability profile evolving
ADT-007	Pan-RAS	Preclinical	—	Better than sotorasib, adagrasib, MRTX1133 (preclinical)	Targets nucleotide-free RAS; preclinical efficacy superior to other agents	No data from clinical studies Does not impact the growth of wild-type RAS PDAC
BI-2865	Pan-KRAS	Preclinical	—	Tumor growth inhibition (preclinical)	Blocks multiple mutants; spares HRAS/NRAS	Compensatory RAS isoform activation which limits effectiveness
YL-17231	Pan-RAS	I	NCT06078800	-	Effective in resistant lines; good PK profile	Early phase, need for further investigation

Abbreviations: PDAC—Pancreatic Ductal Adenocarcinoma; ORR—Objective Response Rate; mPFS—Median Progression-Free Survival; OS—Overall Survival; NSCLC—Non-Small Cell Lung Cancer; TRAEs—Treatment-Related Adverse Events; MW—Molecular Weight; PK—Pharmacokinetics; PROTAC—Proteolysis Targeting Chimera; ctDNA—Circulating Tumor DNA; FDA—U.S. Food and Drug Administration.

## Abbreviations

The following abbreviations are used in this manuscript:

ADM	Acinar-to-ductal metaplasia
AEs	Adverse effects
AGO2	Argonaute 2
AJCC	The American Joint Committee on Cancer
ALK	Anaplastic lymphoma kinase
ALT	Alanine aminotransferase
ARID1A	AT-rich interaction domain 1A
AST	Aspartate aminotransferase
ATM	Ataxia telangiectasia mutated
AuNP	Gold nanoparticles
BCAAs	Branched-chain amino acids
Bcl-2	B-cell leukemia/lymphoma 2 protein
BRCA2	BReast CAncer gene
CCL4	Carbon tetrachloride
CDKN2A	Cyclin-dependent kinase inhibitor 2A
CFPAC-1	Human pancreatic cell line harboring a KRAS G12V mutation
COX2	Cyclooxygenase-2
CRC	Colorectal cancer
ctDNA	Circulating Tumor DNA
CTLA-4	Cytotoxic T-lymphocyte associated protein 4
CXCL8	Chemokine (CXC motif) ligand 8
CXCR2	C-X-C motif chemokine receptor 2
CypA	Cyclophilin A
DCR	Disease control rate
DNA	Deoxyribonucleic acid
dsRNA	Double-stranded RNA
EGF	Epidermal growth factor
EGFR	Epidermal Growth Factor Receptor
EMT	Epithelial-to-mesenchymal transition
ERBB2	Erythroblastic leukemia viral oncogene homologue 2
ERK	Extracellular signal-regulated kinase
ERK1/2	Extracellular signal-regulated kinase 1 and 2
FDA	Food and Drug Administration
FGF3	Fibroblast Growth Factor 3
FGFR3	Fibroblast Growth Factor Receptor 3
GAP	GTPase-activating protein
GATA6	GATA-binding factor 6
G-CSF	Granulocyte colony-stimulating factor
GDP/GTP	Guanosine Diphosphate/Guanosine Triphosphate
GEF	Guanine nucleotide exchange factor
GEFs	Guanine nucleotide exchange factors
GM-CSF	Granulocyte-macrophage colony-stimulating factor
GRB2	Growth factor receptor-bound protein 2
GTPase	Guanosine triphosphatase
HCQ	Hydroxychloroquine
HER2	human epidermal growth factor receptor 2
HRAS	Harvey rat sarcoma viral oncogene
ICIs	Immune checkpoint inhibitors
IFN	Interferon
IL-10	Interleukin-10
IPMN	Intraductal papillary mucinous neoplasms
JNK	Jun N-terminal kinase
KDM6A	Lysine demethylase 6A
KRAS	Kirsten rat sarcoma viral oncogene
LNPs	Lipid nanoparticles
MAPK	Mitogen-activated protein kinase
MDSCs	Myeloid-derived suppressor cells
MHC-I	Major Histocompatibility Complex class I
miRNA	MicroRNA
MLL2	Mixed Lineage Leukemia 2
MMP9	Matrix metalloproteinase-9
mPFS	Median progression-free survival
mTOR	Mammalian target of rapamycin
ncRNA	Non-coding RNA
NET	Neuroendocrine tumor
NF1	Neurofibrin-1
NF-κB	Nuclear Factor kappa B
NR5A2	Nuclear Receptor Subfamily 5 Group A Member 2
NRAS	Neuroblastoma rat sarcoma viral oncogene
NRG1	Neuregulin 1
NSCLC	Non-small cell lung cancer
NTRK	Neurotrophic tyrosine receptor kinase
ORR	Objective response rate
OS	Overall survival
Pan-IN	Pancreatic intraepithelial neoplasia
PARP	Poly(ADP-ribose) polymerase
PC	Pancreatic cancer
PCOS	Polycystic ovarian syndrome
PD-1	Programmed Death 1
PDAC	Pancreatic ductal adenocarcinoma
PD-L1	Programmed Death-Ligand 1
PEI	Pancreatic exocrine insufficiency
PI3K	Phosphoinositide 3-kinase
PI3K	Phosphatidylinositol 3-kinase
PIP3	Phosphatidylinositol (3,4,5)-trisphosphate
PK	Pharmacokinetics
PLGA	Poly9lactic-co-glycolic-acid
PPP	Pentose phosphate pathway
PROTACs	Proteolysis targeting chimeras
PROX1	Prospero Homeobox 1
RAF	Rapid fibrosarcoma
RASA1	p120-RasGAP protein
RASGRF2	RAS protein-specific guanine nucleotide-releasing factor 2
RISC	RNA-induced silencing complex
RNAi	RNA interference
RNF43	Ring Finger Protein 43
SHP2	Src homology 2 domain-containing protein tyrosine phosphatase 2
siRNA	Small interfering RNA
SMAD4	Mothers against decapentaplegic homolog 4
SOC	Standard of care
SOS1/SOS2	Son of sevenless 1 and 2
STING	Stimulators of interferon genes
TAMs	Tumor-associated macrophages
TGF-β1	Transforming Growth Factor beta 1
TIBC	Triple-negative breast cancer
TIGIT	T cell immunoglobulin and immunoreceptor tyrosine-based inhibitory motif domain
TME	Tumor microenvironment
TP53	Tumor protein p53
Tregs	T regulatory cells
TTM	Time to metastasis
UBL4A	Ubiquitin-like protein 4A
VEGF	Vascular endothelial growth factor
